# Anatomical reconstruction of coracoclavicular and acromioclavicular ligaments using an autologous tendon graft provides excellent outcomes in acute acromioclavicular joint dislocation

**DOI:** 10.1002/ksa.70051

**Published:** 2025-08-29

**Authors:** Efstathios Konstantinou, Alexandros Koskiniotis, Nikolaos Stefanou, Antonios Koutalos, Efstratios Athanaselis, Michael Hantes, Socratis Varitimidis

**Affiliations:** ^1^ Department of Orthopaedic Surgery & Musculoskeletal Trauma University Hospital of Larissa, School of Health Sciences University of Thessaly Larissa Greece

**Keywords:** acromioclavicular, acute, autograft, dislocation, reconstruction

## Abstract

**Purpose:**

Common surgical techniques for managing acute acromioclavicular (AC) injuries include reconstruction of the coracoclavicular (CC) ligaments using tendon grafts or high‐strength artificial looping materials, as well as fixation with a hook plate. This study presents a thorough analysis of the outcomes of anatomical reconstruction of both the CC and AC ligaments using a single‐strand semitendinosus tendon graft.

**Methods:**

All patients with acute AC joint dislocation who underwent anatomical reconstruction of the CC and AC ligaments between 2017 and 2022 were included in this retrospective analysis. Postoperative evaluation of clinical and functional outcomes was conducted using the Simple Shoulder Test, QuickDASH and Constant–Murley scores. Radiographic assessments were used to determine any loss of reduction.

**Results:**

The study included twelve male patients with a mean age of 37.8 years (range: 23–64). According to the Rockwood classification, three patients had Type III, three had Type IV, and six had Type V dislocations. All patients underwent anatomical reconstruction of the CC and AC ligaments. The most recent follow‐up, with a mean duration of 31.8 months (range: 12–64 months), demonstrated excellent postoperative functional outcomes, with scores of 87 (SD = 4.1) for the Simple Shoulder Test, 2.9 (SD = 4.5) for QuickDASH and 89.7 (SD = 3.1) for the Constant‐Murley score. Radiographic assessment showed a reduction in CC distance from a preoperative mean of 16.6–8.9 mm at final follow‐up. Partial loss of reduction was noted in two patients; however, neither exhibited functional impairment or activity limitations that required surgical revision. Minor complications included wound dehiscence (one patient) and persistent numbness at the incision site (two patients).

**Conclusions:**

Reconstruction of the CC and AC ligaments using an autologous semitendinosus tendon graft for acute AC joint dislocation results in excellent clinical outcomes and satisfactory radiographic findings.

**Level of Evidence:**

Level IV.

**Abbreviations**
AC: acromioclavicular
ACJ: acromioclavicular joint
CC: coracoclavicular
CT: computed tomographic
MRI: magnetic resonance imaging
PROM: Patient Reported Outcome Measure
SD: standard deviation

## INTRODUCTION

Acromioclavicular joint (ACJ) dislocations account for approximately 9% of shoulder girdle injuries [5]. Studies indicate that this type of injury is more prevalent among male patients, particularly in their third decade of life [15]. The most common mechanism of ACJ dislocation involves a direct force applied to the superior aspect of the acromion during a fall in which the patient extends his upper extremity in order to protect himself [17]. The severity of ACJ dislocations is classified using the Rockwood classification, which ranges from Type I to VI based on radiographic evaluation [18]. While Grade I and II dislocations are typically managed nonoperatively, Grades IV, V and VI generally require surgical intervention [10]. However, the optimal treatment strategy for Grade III ACJ dislocations remains a topic of debate [12].

When surgical management of acute ACJ dislocation is indicated, a plethora of techniques are available [22]. These techniques span from reduction and fixation of the ACJ to reconstruction of the acromioclavicular (AC) and coracoclavicular (CC) ligaments. Traditional methods, such as the Weaver–Dunn procedure and the Bosworth screw, are now used less frequently, while newer approaches, including anatomical reconstruction of the CC ligaments and the use of endobuttons or TightRope systems, are gaining popularity. However, despite the increasing volume of comparative studies, the potential advantages of arthroscopic surgery remain uncertain [24].

Anatomic ACJ reconstruction was demonstrated to provide better results in chronic dislocations compared to other techniques, as highlighted by recent studies [19, 26]. A hardware‐free anatomic reconstruction technique using a semitendinosus tendon graft was described by Saccomanno et al. [20, 21] for the treatment of chronic ACJ dislocations. However, this technique has never been validated in the context of acute dislocations. The purpose of this study was to evaluate mid‐term clinical and radiological outcomes of the technique proposed by Saccomanno et al. [20] for anatomical reconstruction of the CC and AC ligaments using a single‐strand semitendinosus tendon graft. However, unlike Saccomanno's original application for chronic ACJ dislocations, this study applied the technique in the setting of acute ACJ dislocations. Our hypothesis is that this hardware‐free technique will achieve satisfactory clinical and radiographic outcomes with minimal complications.

## MATERIALS AND METHODS

This retrospective study was approved by the institutional review board (IRB No. 24474/07.06.24). A case‐series analysis included all patients who underwent ACJ reconstruction following an acute ACJ dislocation at our institution between January 2017 and December 2022. A written informed consent was signed by each patient before entering the study. Inclusion criteria included acute AC joint dislocations of Types III–V, age 18 years or older at the time of surgery, a minimum follow‐up of one year, and a complete radiographic examination, including AP and Zanca views of both clavicles. Additional criteria comprised AC joint instability on clinical examination accompanied by symptoms such as persistent pain, weakness, loss of shoulder flexion and/or abduction, either at the initial presentation or within seven to 14 days post‐injury, unless surgery had already been performed during that time. To determine the type of ACJ dislocation according to the Rockwood classification, additional computed tomography (CT) was performed. Patients were excluded if they had concomitant shoulder pathology, degenerative changes of the AC or glenohumeral joint, joint infections, or concurrent neurological diseases. Additionally, patients who underwent gracilis tendon harvesting were excluded. Two authors (E.K. and A.K.) independently identified eligible patients through a computerised database of surgeries for shoulder pathology at our department, and the medical records were reviewed to gather all relevant data.

Clinical examination and radiological evaluation were performed to diagnose acute ACJ dislocations. Clinical re‐evaluation of the acute injury was conducted 7–14 days post‐injury to reduce the impact of acute pain and swelling and to draw more reliable conclusions regarding scapular retraction, glenohumeral range of motion, and rotator cuff strength. The radiological protocol included plain radiographs and CT imaging for diagnosis and to evaluate relative bone positions or rule out fractures of the distal clavicle, acromion, or coracoid process. Magnetic resonance imaging (MRI) was not routinely used unless there was strong clinical evidence of intra‐articular shoulder pathology or rotator cuff injury.

All patients followed an individualised rehabilitation programme aimed at restoring functional impairments, either until surgery or until their decision for non‐surgical management. A minimum follow‐up period of one year was required for all patients. Preoperative data were extracted from medical records, while postoperative outcomes were assessed at the most recent follow‐up using patient‐reported outcome measures (PROMs). Clinical and functional outcomes were evaluated using the Simple Shoulder Test, QuickDASH and Constant‐Murley scores. Additionally, radiographic examinations were performed to detect any potential loss of reduction.

**Figure 1:**
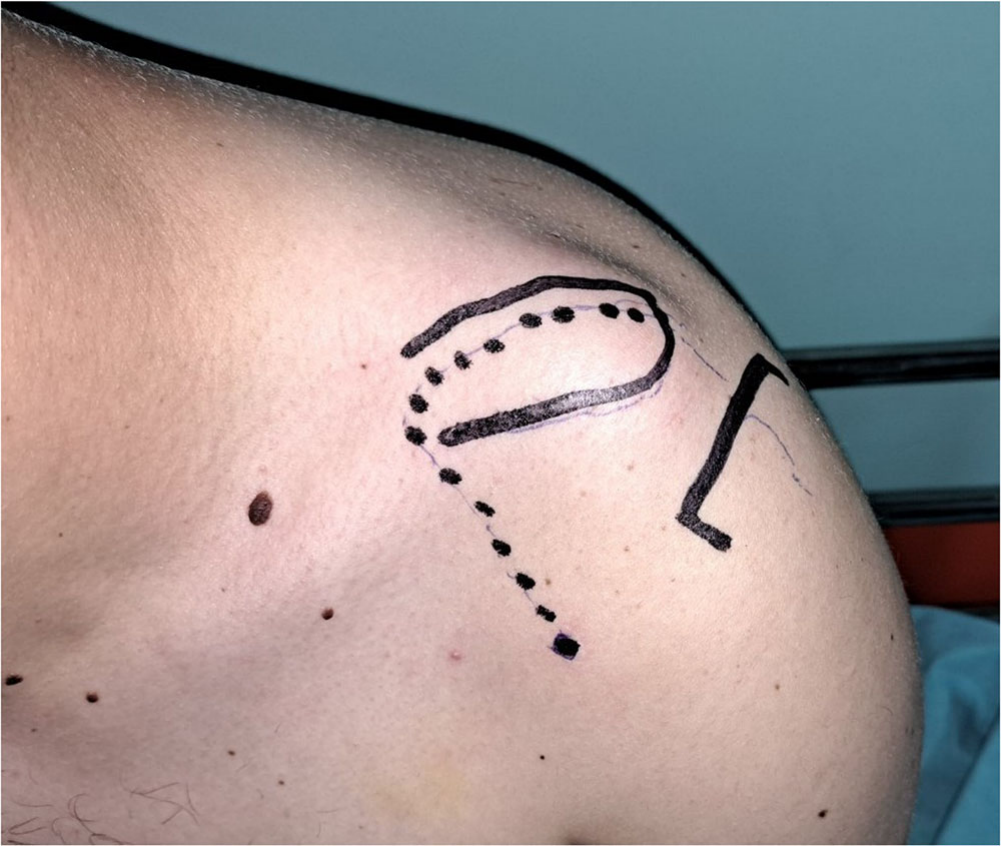
The planned skin incision is marked by the dotted line. The distal clavicle and the acromion are outlined to guide surgical exposure.

The surgical procedure we perform is based on the technique proposed and thoroughly detailed by Saccomanno et al. [20]. The incision is designed to provide optimal exposure to the superior aspect of the clavicle, the AC joint, and the anterior third of the acromion (Figure 1).

**Figure 2:**
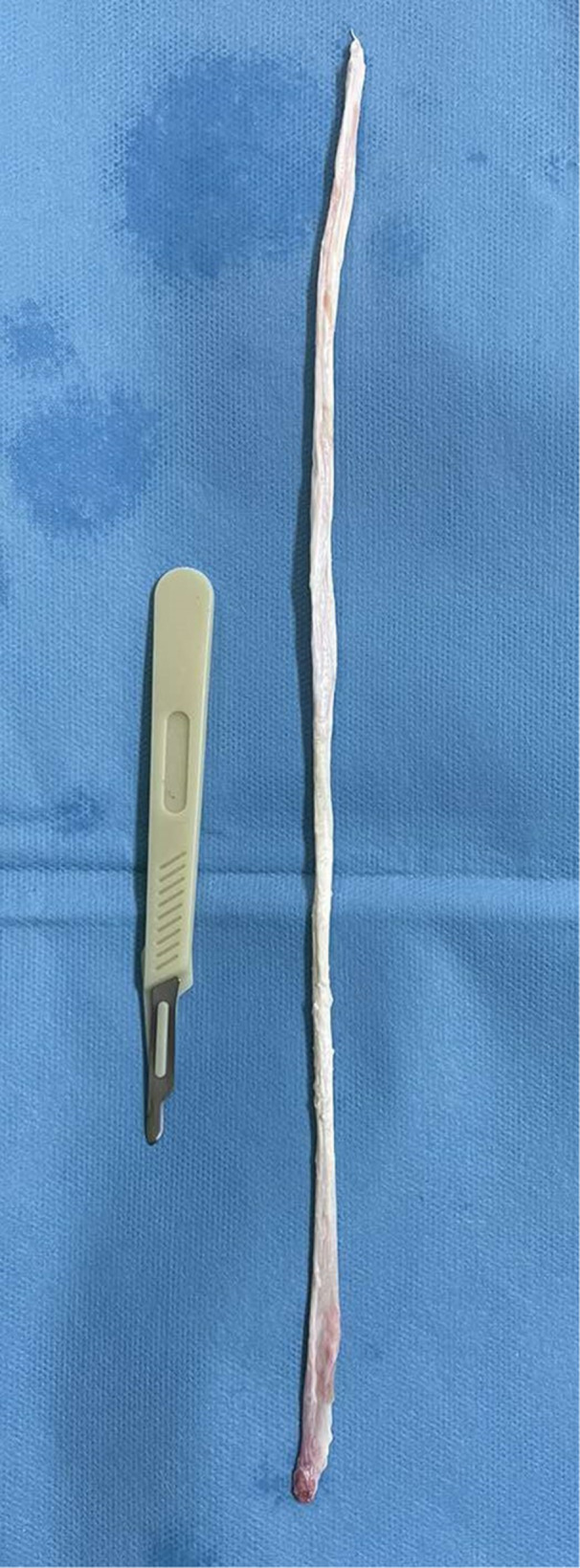
Single‐strand semitendinosus tendon autograft.

The AC joint reconstruction involves using a single‐strand semitendinosus tendon autograft (Figure 2).

**Figure 3:**
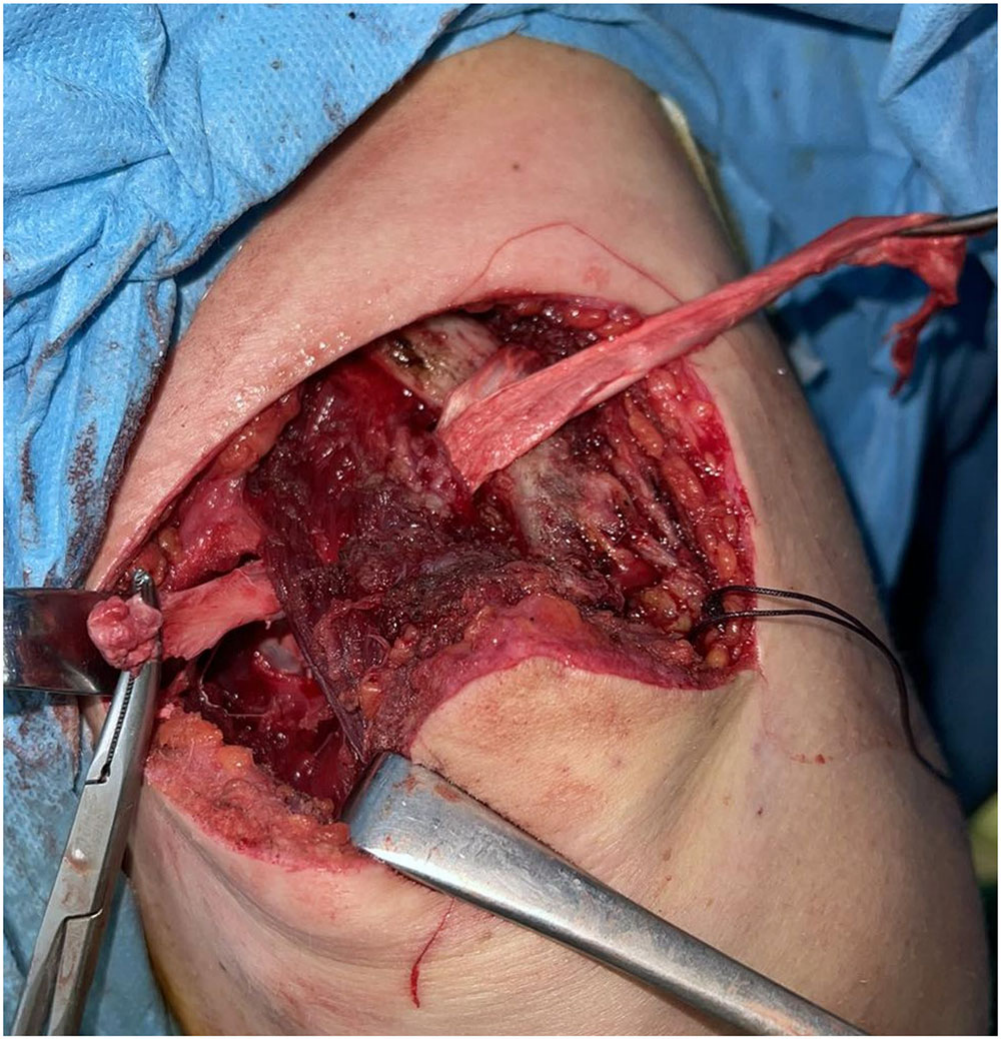
The graft is passed underneath the coracoid process and through two bone tunnels in the clavicle.

This graft is passed underneath the coracoid process and through two bone tunnels in the clavicle (4.5 mm in diameter and at least 1–1.5 cm distance between them in order to avoid a clavicle fracture) corresponding to the native attachment sites of the conoid and trapezoid ligaments (Figure 3).

**Figure 4:**
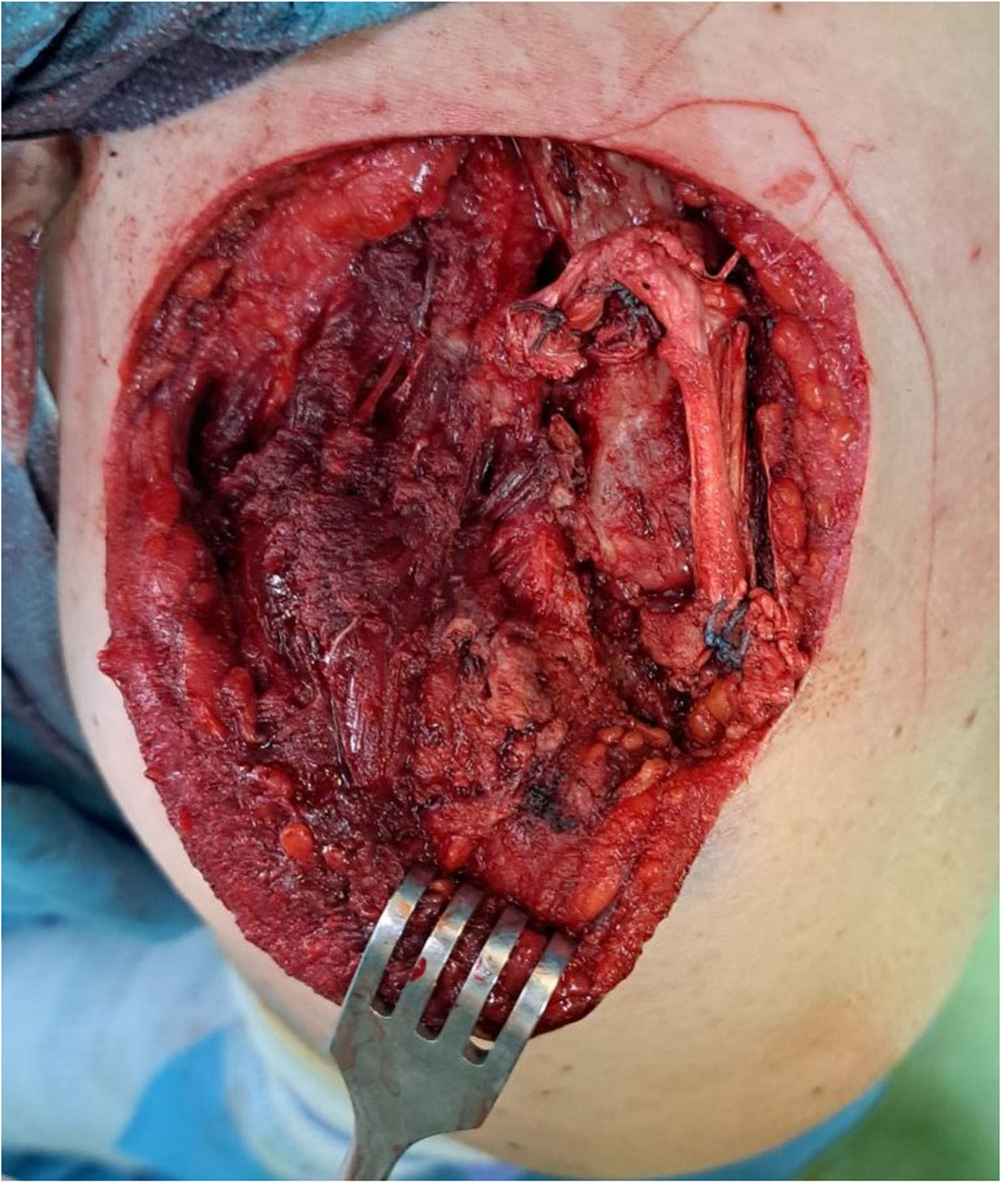
The graft is looped through a tunnel in the acromion, recreating the superior and inferior acromioclavicular (AC) ligaments.

Additionally, a tunnel was created in the acromion, allowing the lateral end of the graft to be looped from top to bottom, effectively recreating the superior and inferior AC ligaments (Figure 4).

Unlike the original technique, we did not employ non‐biological fixation to augment the reconstruction. Patients were administered low molecular weight heparin for 3 weeks postoperatively due to the use of tourniquet for graft harvesting. Patients were not subjected to any restrictions on knee movement postoperatively.

All patients followed a standardised postoperative protocol, which included wearing a sling with the upper limb in internal or neutral rotation for 6 weeks. From the 3rd to 6th weeks, the rehabilitation programme focused on achieving full‐range passive motion. After 6 weeks, the protocol allowed for active forward flexion, muscle strengthening, proprioception and plyometric exercises, continuing until the tenth postoperative week. Patients were permitted to return to full activity between 4 and 6 months postoperatively, depending on their specific requirements.

### Statistics

Statistical analysis was performed using SPSS software (version 26 for Windows). The dataset included preoperative and postoperative functional scores (QuickDASH, Constant‐Murley and Simple Shoulder Test), affected limb, Rockwood classification, trauma mechanism, duration of surgery and time from admission to surgery for each patient. The Shapiro–Wilk test was used to confirm the normal distribution of all data. Mean functional scores assessed at the two time points (preoperative and final postoperative follow‐up) were compared using paired *t*‐tests, with a significance level set at *p* < 0.05. Radiographic evaluation was conducted to assess any potential loss of reduction.

## RESULTS

A total of 12 male patients were included in the study, all of whom were eligible, with no patients lost to follow‐up. The right upper extremity was the most frequently affected (83%, 10/12). The mean age was 37.8 years, with falls on an outstretched arm being the most common mechanism of injury, followed by motorcycle accidents. According to the Rockwood classification, three cases were Type III, three were Type IV, and six were Type V. The decision for surgical intervention in Type III dislocations was influenced by patients' engagement in heavy labour. The mean time to surgery was 9.3 days (range: 7–14), with a median operative duration of 94 min.

All patients were discharged within a maximum of 3 days of hospitalisation. Function significantly improved, as indicated by pre‐ and postoperative scores at a mean follow‐up of 31.8 months (range: 12–64 months). Notably, the mean Constant‐Murley score increased from 17.6 ± 3.5 preoperatively to 89.7 ± 3.1 postoperatively (*p* < 0.001), the Simple Shoulder Test improved from 6.1 ± 6.3 to 87 ± 4.1 (*p* < 0.001), and the QuickDASH score significantly decreased from 64 ± 4.3 to 2.9 ± 4.5 (*p* < 0.001) (Table 1).

**Table 1:** Results. Functional scores showed significant improvement postoperatively.

PROMs	Mean preoperative values	Mean postoperative values	*p*‐Value
QuickDash	64 (SD = 4.3)	2.9 (SD = 4.5)	**<0.001**
Constant‐Murley	17.6 (SD = 3.5)	89.7 (SD = 3.1)	**<0.001**
Simple Shoulder Test	6.1 (SD = 6.3)	87 (SD = 4.1)	**<0.001**

Abbreviations: PROMs, Patient‐Reported Outcome Measures; SD, standard deviation.

**Figure 5:**
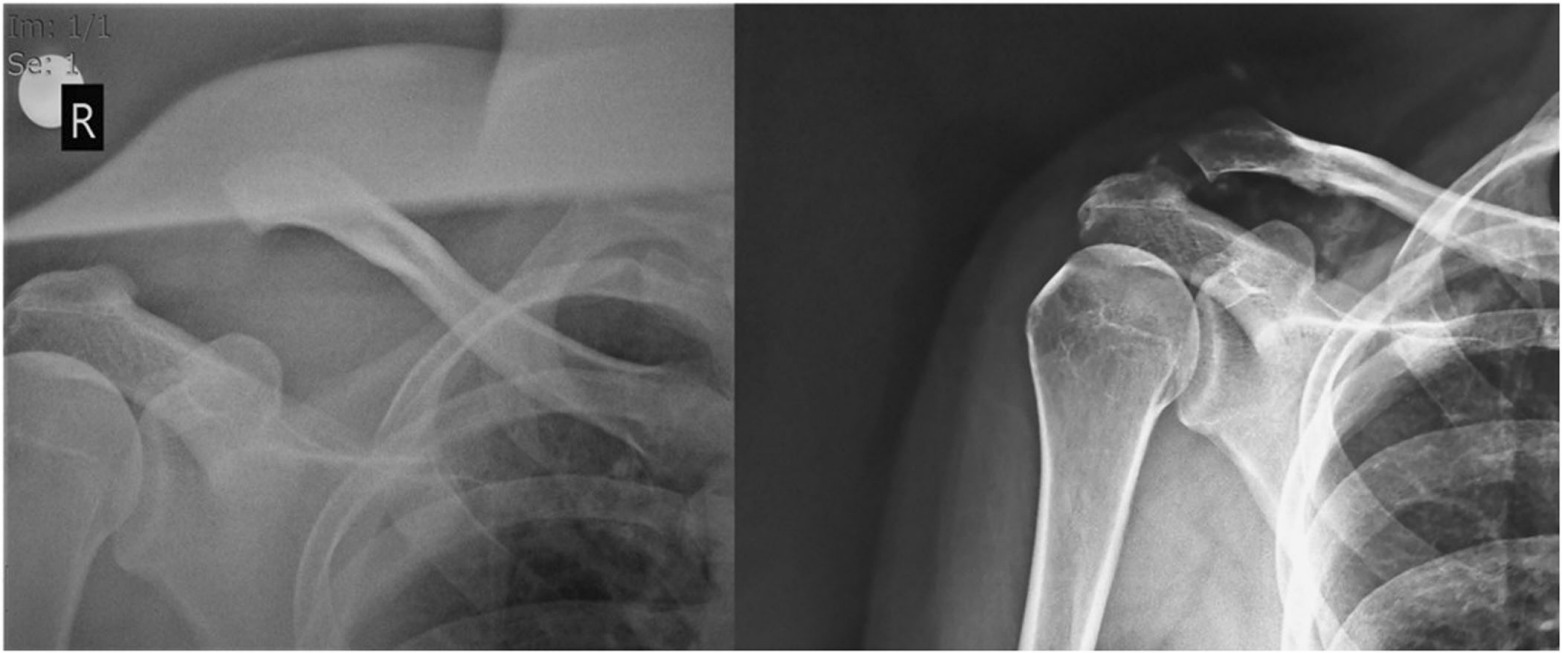
27‐years‐old patient preoperatively and 18 months postoperatively.

The mean time to return to work was 9.7 weeks. Two patients experienced a partial loss of reduction during follow‐up, with one patient admitting to non‐compliance with postoperative instructions and returning to work just 4 weeks postoperatively (Figure 5). However, neither of these patients exhibited functional impairments or activity limitations that required surgical revision.

**Figure 6:**
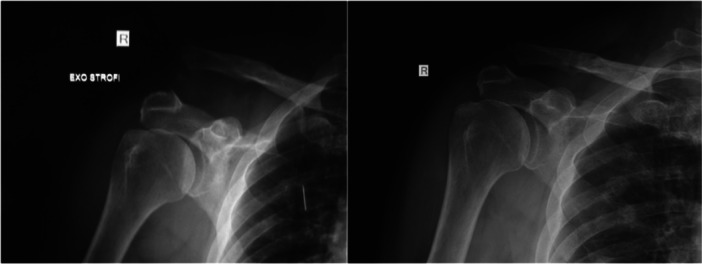
64‐years‐old patient preoperatively and 13 months postoperatively.

Radiological assessment of reduction was performed by measuring the CC distance preoperatively, postoperatively, and at follow‐ups. The mean CC distance on the unaffected side was 7.9 mm (standard deviation [SD] 0.7). Preoperatively, the mean CC distance on the affected side was 16.6 mm (SD 3.7). The immediate postoperative mean CC distance was 8 mm (SD 0.8), which increased to 8.9 mm (SD 1.9) at the final follow‐up (*p* < 0.001) (Figure 6).

Minor complications, such as persistent numbness at the incision site (two patients) and wound dehiscence (one patient), were observed. No donor site morbidity related to semitendinosus tendon harvesting was observed in any patient.

## DISCUSSION

This study demonstrates that anatomical reconstruction of the CC and AC ligaments using an autologous tendon graft, without the use of hardware, results in excellent clinical and satisfactory radiological outcomes at mid‐term follow‐up for acute ACJ dislocations. The surgical technique applied in this study was originally proposed for chronic cases and has been shown to yield favourable clinical and radiological outcomes with a minimum follow‐up of 2 years [21].

When discussing ACJ dislocation, it is important to distinguish between acute cases (occurring within 3 weeks of injury) and chronic cases (occurring more than 3 weeks post‐injury) [8, 30]. This distinction is crucial because the AC ligaments begin to lose their healing potential after 3 weeks. Therefore, it is imperative that acute high‐grade dislocations, when indicated, are treated surgically without delay [3, 13]. In many cases, even in the acute phase, especially in Types III–V, it seems that there are either insufficient ligamentous remnants for repair or that an excessive inflammatory process alters the healing dynamics, particularly in borderline cases that transition from the acute phase to the chronic phase (10–21 days). Since this is a reconstruction technique, it is not affected by whether healing potential of the native ligaments exists or not, thereby reducing treatment failure rates.

A wide range of surgical techniques has been proposed for managing acute ACJ dislocations; however, the optimal approach remains undetermined, presenting surgeons with a significant therapeutic challenge [27]. Modern anatomical CC ligament reconstruction techniques are increasingly favoured as they address both vertical and horizontal instability [24]. This preference is further supported by recent biomechanical studies, which demonstrate that anatomical reconstructions using free grafts provide superior stability and healing potential compared to alternative ligament transfer methods [27].

When selecting a graft, the initial challenge lies in choosing between a synthetic or biological option. One advantage of synthetic grafts, particularly if readily available, is their ease of application. They eliminate the risk of infection transmission and can enhance healing, thereby accelerating rehabilitation and facilitating a quicker return to normal activities [25]. However, the use of synthetic grafts has declined due to well‐documented soft tissue reactions and the high revision rates reported in the literature [14, 24].

Regarding the selection of biological grafts, the literature remains inconclusive on whether autografts or allografts are preferable for anatomical reconstruction of the CC and AC ligaments following acute injury [24, 27]. A recent review on graft selection for chronic ACJ instability found that both autografts and allografts provide comparable outcomes and are reliable options for managing this condition [9]. However, as with synthetic grafts, a key limitation is the lack of allograft availability in certain countries or hospitals and the low cost‐effectiveness ratio.

Advancements in arthroscopy have the potential to improve clinical outcomes and allow patients to return to their daily activities more quickly [7]. Although various arthroscopic and mini‐open arthroscopically assisted techniques are available, and an increasing number of studies compare these approaches to open surgery, there is currently insufficient evidence to confirm the superiority of arthroscopy [24]. However, arthroscopy is likely to play a growing role in managing high‐grade ACJ dislocations, particularly given the potential for identifying concomitant intra‐articular pathologies that might otherwise go unnoticed and inadequately treated [2]. Nonetheless, ongoing vigilance is required regarding the complications associated with arthroscopy. While infection and neurovascular compromise may not be of primary concern, the rates of fractures and loss of reduction remain significant challenges [27].

All surgical procedures carry inherent risks, including superficial wound infection, skin irritation, or wound dehiscence; thus, appropriate precautionary measures are essential. By employing this technique, we eliminate the possibility of commonly reported complications such as hardware failure and subacromial osteolysis, which are typically associated with the use of hook plates [1, 6, 11, 31]. A notable concern in CC ligament reconstruction techniques is the risk of coracoid fractures. The technique used in this study involves passing the tendon graft beneath the coracoid without the need for drilling, thereby mitigating this fracture risk. According to Saccomanno et al., the originator of this technique, it also eliminates hardware‐related complications, such as hardware failure, the need for hardware removal, osteolysis, and late clavicle fractures [21]. Another potential advantage of avoiding synthetic fixation is a reduced risk of clavicular tunnel widening, which has been associated with implant type and postoperative loss of reduction [4, 29].

Finally, our concern was the varying degrees of reduction loss observed during the most recent radiological evaluations. However, as documented in previous studies [16, 23, 28], we found no correlation between loss of reduction and patients' clinical outcomes. It is important to emphasise that the reduction loss was identified through radiographic examination and was not reported by patients as an aesthetic concern; moreover, there was no need for additional surgical intervention.

## LIMITATIONS

This study has several limitations. It was conducted as a retrospective case‐series study, with preoperative clinical findings obtained accordingly. Selection bias is inherent, as patients were chosen for this procedure based on the surgeon's preference, and cases where the gracilis tendon was harvested were excluded.

Only a single surgical technique was assessed, without a control group for comparison. Preoperative PROMs were recorded immediately after the injury, which may have led to an overestimation of baseline scores. Consequently, the significant improvement in clinical scores may be slightly overstated.

Due to the rarity of cases requiring ACJ reconstruction, the study sample size was small, resulting in limited statistical power and restricting the generalisability of our conclusions. However, with a mean follow‐up of 31.8 months, this study offers one of the longest mean follow‐ups in the current literature for a surgical technique utilising an autologous tendon graft without hardware.

## CONCLUSIONS

Treatment of acute AC joint dislocations by reconstructing the CC and AC ligaments using a semitendinosus tendon graft results in excellent clinical and satisfactory radiological outcomes. This surgical technique proved to be an effective option for managing high‐grade AC dislocations without the need for hardware.

## AUTHOR CONTRIBUTIONS

All listed authors have contributed substantially to this work. Literature search, study design, and primary manuscript preparation were performed by Efstathios Konstantinou, Alexandros Koskiniotis, and Nikolaos Stefanou. Data collection, analysis, and interpretation of results were performed by Efstathios Konstantinou and Alexandros Koskiniotis. Statistical analysis was performed by Efstathios Konstantinou and Alexandros Koskiniotis. Final manuscript drafting and editing were performed by Efstathios Konstantinou, Nikolaos Stefanou, Antonios Koutalos, Efstratios Athanaselis, Michael Hantes and Socratis Varitimidis.

## CONFLICT OF INTEREST STATEMENT

The authors declare no conflicts of interest.

## ETHICS STATEMENT

This study was approved by IRB number: 24474/07.06.24 by the Ethics Committee of University Hospital of Larissa. A written informed consent was signed by each patient before entering the study. The authors affirm that human research participants provided informed consent for publication of the images in Figures 1, 2, 3, 4, 5, 6.

## Data Availability

Anonymised data from the study are available upon reasonable request.
